# Occupational accidents in the Danish merchant fleet and the nationality of seafarers

**DOI:** 10.1186/s12995-014-0035-4

**Published:** 2014-10-23

**Authors:** Balázs Ádám, Hanna Barbara Rasmussen, Randi Nørgaard Fløe Pedersen, Jørgen Riis Jepsen

**Affiliations:** Centre of Maritime Health and Society, Institute of Public Health, Faculty of Health Sciences, University of Southern Denmark, Niels Bohrs Vej 9-10, Esbjerg, DK-6700 Denmark; Institute of Public Health, College of Medicine and Health Sciences, United Arab Emirates University, P.O. Box 17666, Al Ain, United Arab Emirates

**Keywords:** Maritime health, Occupational safety, Injury rate, Nationality differences, Safety culture

## Abstract

**Background:**

The aim of the study was to examine occupational accidents reported from non-passenger merchant ships registered in the Danish International Ship Register in 2010-2012, with a focus on analysing nationality differences in the risk of getting injured in an accident.

**Methods:**

Data about notified occupational accidents were collected from notifications sent to the Danish Maritime Authority and from records of contact with Danish Radio Medical. Events were matched by personal identification and accident data to create a unified database. Stratified cumulative time spent on board by seafarers was used to calculate accident rates. Incidence rates of different nationalities were compared by Poisson regression.

**Results:**

Western European seafarers had an overall accident rate of 17.5 per 100000 person-days, which proved to be significantly higher than that of Eastern European, South East Asian and Indian seaman (adjusted incidence rate ratio 0.53, 0.51 and 0.74, respectively), although differences decreased over the investigated period. Smaller but in most cases still significant discrepancies were observed for serious injuries. The back injury rate of Western European employees was found especially high, while eye injuries seem to be more frequent among South East Asian workers.

**Conclusions:**

The study identified substantial differences between nationalities in the rate of various accidents reported from merchant ships sailing under the Danish flag. The differences may be attributed to various factors such as safety behaviour. Investigation of special injury types and characterisation of effective elements of safety culture can contribute to the improvement of workplace safety in the maritime sector.

## Background

Denmark has one of the largest merchant fleets in the world. In 2012 there were over 600 Danish-flagged ships sailing internationally, out of which 400 were cargo vessels [1]. These ships are registered in the Danish International Ship Register. Crews of registered non-passenger merchant ships are typically of international composition, including workers from all over the world, but mainly from Denmark, the Philippines, India and Poland. Although workplace health and safety requirements are the same, employees’ attitude toward workplace safety and the risk of getting injured on board can vary by nationality.

The challenges relating to the special work environments and activities at sea imply an increased level of risk for being involved in an occupational accident in general. Although maritime workplace safety is improving, seafaring is still associated with high rates of occupational accidents, compared with other occupational settings. According to the findings of Roberts and Hansen, the rate of fatal occupational accidents among seafarers serving on British merchant ships was more than twenty times higher than that for all workers in Great Britain in the period 1986-1995 [2]. Increased risks were also recorded in Denmark at the same time. Hansen et al. observed that a seafarer on board a Danish merchant ship had an 11.5 times higher risk of a fatal workplace accident than Danish male workers on shore [3]. As a similar analysis pointed out later that although the situation had improved the difference remained high, at more than six-fold, between 2002 and 2009 [4]. In addition to the higher rate of fatal accidents, a high frequency of non-fatal occupational accidents can also be observed. In a multinational study involving seafarers from 11 countries, the seafarers were asked about occupational injuries that had happened during their last tour of duty. More than 9% reported an injury during their last voyage and almost half of them had sustained an injury leading to at least one day of incapacity [5].

Multiple factors can contribute to the occurrence of a workplace accident, such as the safety behaviour of employees, job activities, workplace conditions, safety measures and management systems in place. Human factors play an especially important role in the causation of occupational accidents [6]. The safety behaviour of an employee is determined by several factors, such as individual physical and mental conditions, as well as by various aspects of the social and cultural context in which these people grew up and manage their lives.

The current study investigates occupational accidents and the resulting injuries as reported from ships registered in the Danish international merchant fleet in the period 2010-2012. The main focus of the study is to analyse whether the seafarers’ nationality influences their risk of getting involved in an accident. The study discusses special types of occupational injuries and attempts to explain the observed differences between nationalities. This question is highly relevant when taking into consideration the wide-scale representation of different nationalities in the international seafaring workforce.

## Methods

### Data sources

This correlational study used two sources to acquire data about accidents that happened on non-passenger merchant ships registered in the Danish International Ship Register (DIS) in 2010-2012. The paper-based notifications of occupational accidents reported to the Danish Maritime Authority (DMA) provided one source. In accord with the requirements specified in the Prevention of Accidents (Seafarers) Convention of 1970 (No. 134) and the Prevention of Accidents (Seafarers) Recommendation of 1970 (No. 142), adequate reporting of maritime occupational accidents must be ensured. Accidents occurring on board of ships registered in the DIS that cause at least one day away from work beyond the day of accident (lost time accident, LTA) or inability of the injured person to carry out the usual work activity for one day or more in addition to the day of the injury (restricted work accident, RWA) must be reported to the DMA. Besides the mandatorily notified cases, milder injuries are often reported to the authority as well. The following identification data, exposure and outcome information were extracted from the reports: the personal identification number, name, gender, position and nationality of the injured person; the ship’s name, call sign and company; the date of accident, the description of the event and the injury; the number of work-days lost, the need to seek medical help and the treatment administered.

Documents about contacts with Radio Medical Denmark (RMD) for medical advice in relation to an injury that happened on board DIS-registered ships during the period 2010-2012 were the other information source. The information media varied from official forms to emails exchanged between the communicating parties. The RMD records were manually matched with the records from the DMA using personal identification numbers and names of injured seafarers, as well as dates and descriptions of accidents that were typically documented in both data sources. In the Danish registration system citizens possess a 10-digit unique identifier and foreign employees signed up for work on a ship under the Danish flag are also provided with a personal identification number that, together with names, could be used effectively for finding identical cases. Ethical approval for data handling was obtained from the Danish Data Protection Agency.

Person-time at risk was used to characterise the source population of cases and to calculate the accident risk. All dates for signing on and off a ship are notified to the DMA. Consequently, the Authority’s database could provide us with information about time spent on board DIS-registered non-passenger ships in the investigated period. The analysis used the cumulative number of days spent on board, stratified by year of sign-on, nationality, ship’s size, employee’s work status and age.

### Variables

All occupational accidents occurring on a DIS-registered non-passenger merchant ship in the period 2010-2012 were included in the study. An accident was considered occupational if it happened to a seafarer while on board. Events that happened onshore were excluded. Only notified cases of ill-health that were caused by a sudden health-damaging event were considered occupational injuries, so occupational diseases were excluded. Injuries caused by any forms of inter-personal violence including pirate attacks were likewise excluded from the analysis.

Ships were categorised by size and type. They were divided into two size categories, those ≥3000 gross tonnage and those below. Various ship types were recorded in the database, but the analysis covered only accidents reported from non-passenger merchant ships. The reasons for the restriction were the high heterogeneity of nationalities and the availability of reliable data on the time spent on board, which were only assured for the crews of non-passenger ships.

Employees were grouped by occupation, as officer or non-officer. An official list detailing position categories in the maritime sector was used to transcribe positions on board in a systematic manner. Navigation officers, including masters, and engineer officers were categorised as officers. All other positions including any catering and service positions were regarded non-officers.

The age of the employees was calculated based on the first 6 digits of their personal identification number and the date of the accident. The analysis handled age in 10-year age groups (16-25, 26-35, 36-45, 46-55, 56-65, >65).

Nationalities were allocated into four categories. Western European seafarers included citizens of Western European countries bordered to the east by Norway, Finland, Germany, Austria and Italy, plus Greece and Cyprus. The majority of this group were Danish seafarers. Eastern European employees were those from Central and Eastern European countries, mainly from Poland. South East Asian seafarers formed the second largest group after Western Europeans. This category was deemed to cover the Philippines, Vietnam, Laos, Cambodia, Thailand, Myanmar, Singapore, Malaysia, Brunei and Indonesia. In practice this group comprised Filipino employees. Indian seafarers formed a separate group in the study due to their high number and distinct cultural background from South East Asian people. The remaining seafarers represented six further countries from all over the world. Due to their diverse backgrounds and relatively low number they were not included in the nationality comparison.

Analyses were carried out on all injuries that fulfilled the inclusion criteria and additionally on three special categories: serious injuries, back injuries and eye injuries. A serious injury had to be notified as a LTA and/or indicated as involving incapacitation of at least one day beyond the day of accident. A case was also considered serious if it required medical treatment or involved contact with Danish Radio Medical. Back and eye injuries were recognised if there was a clear indication of damage to these specific body parts.

### Statistical analysis

Incidence rates of occupational accidents per 100000 person-days spent on board were determined. Cumulative data for the examination period were used to calculate incidence rate ratios for each nationality group, using the rate of Western European seafarers as reference value. Poisson regression analysis was carried out to test for significant differences in between strata. The statistical significance was defined on 5% significance level. Apart from the crude comparison, analyses were also performed following adjustment for potential confounding factors that were identified in the study as having influence on the accident rate, that is ship size, position and age of employee. Analyses were also conducted on a year-by-year basis in the same way as completed with cumulative data. The statistical analysis was performed using Stata/IC version 12.1 statistical software.

## Results

Altogether 1336 cases occurring on DIS-registered non-passenger merchant ships were reported to the Danish Maritime Authority or led to contact with Danish Radio Medical during the three year study period. 28 injuries acquired on shore, 11 occupational diseases and 23 injuries caused by pirate attacks and other forms of violence were excluded from the analysis.

The majority of cases were derived from notifications sent to the DMA. Out of 1274 included injuries, 881 (69%) were only reported to DMA, while 267 cases sought medical advice from Radio Medical. The remaining 126 cases were notified to the DMA and involved calls to RMD, thus providing information about the accidents from both sources.

The absolute number of reported accidents showed slight increase over the three-year study period. The number grew from 383 in 2010 to 435 in 2011 and 456 in 2012. The majority of injured workers were from Western Europe and South East Asia, with 582 (46%) and 389 (31%) cases in the entire period, respectively.

### Accident rates

**Figure 1:**
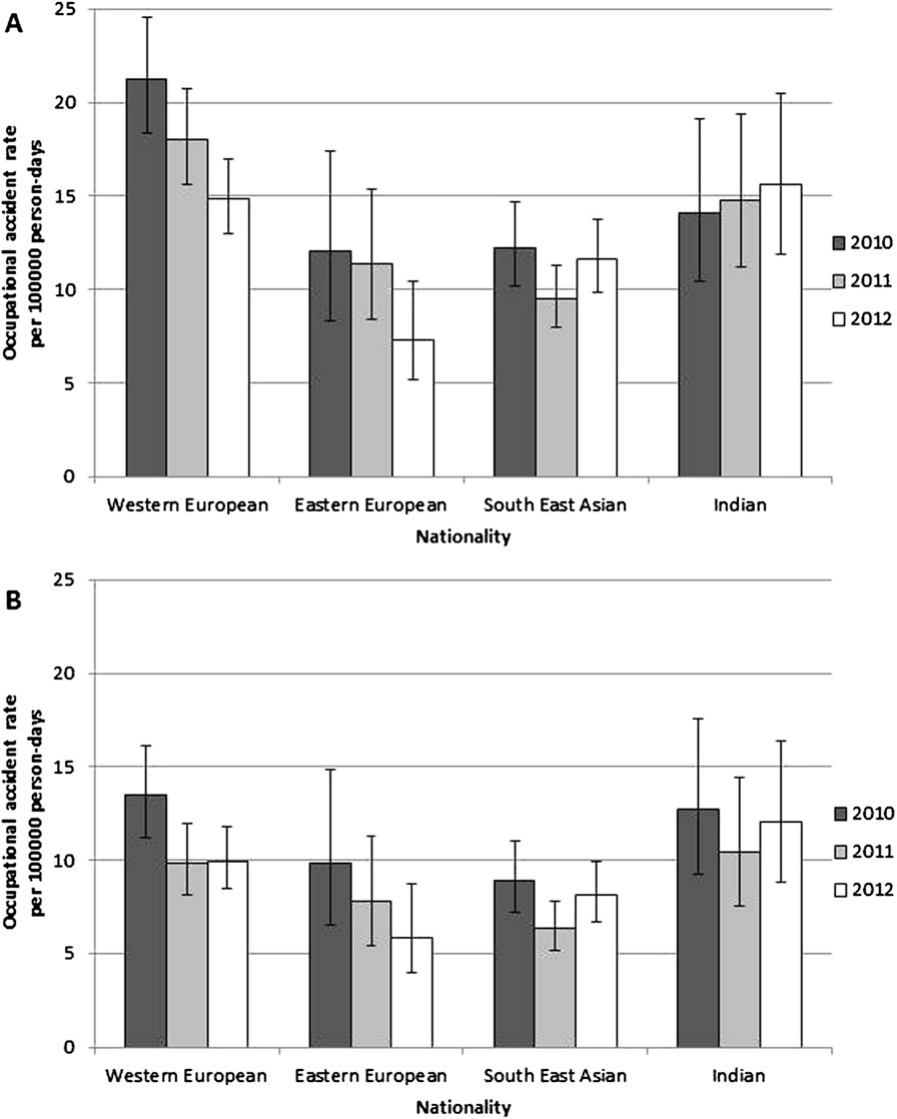
**Accident rate of all (A) and serious (B) occupational injuries reported to the Danish Maritime Authority and to Radio Medical Denmark by nationality of seafarers working on non-passenger merchant ships registered in the Danish International Ship Register.**

The incidence rate of occupational accidents among the different nationality groups over the three years investigated shows diverse pattern. The rate was high among Western European seafarers but followed a remarkable decreasing trend in the examined period (Figure 1A). Accidents were considerably less frequent among Eastern European and South East Asian employees. Eastern European seafarers experience a reduction similar to Western Europeans, while the trend among South East Asians was inconclusive. Indian workers occupied an intermediate position among the nationality groups and their accident rate increased slightly over the period. The results show unequivocally low injury rates among South East Asian and Eastern European seafarers compared with their Western European colleagues (Table 1). The difference was significant both in the crude and adjusted analysis and was also observed in each year separately (detailed statistics on annual data are not shown). Indian workers showed a reduced risk compared with Western Europeans. Crude comparison determined a difference just not significant, but it gained significance after adjustment for confounding factors. When the analysis was performed by years, Indian seafarers had a significantly lower injury rate in 2010 and 2011 but not in 2012.

**Table 1:** **Relationship between the nationality of seafarer and the rate of occupational accidents that occurred on board non-passenger merchant ships registered in the Danish International Ship Register in 2010-2012**

**Nationality**	**Accidents**	**Person-days**	**Incidence/100000 days**	**Incidence rate ratio**	**95% CI**	**Incidence rate ratio**	**95% CI**
				**Crude**		**Adjusted** ^**a**^	
*All injuries*							
Western European	582	3325410	17.50	1		1	
Eastern European	101	1026072	9.84	0.56	0.45 to 0.69	0.53	0.43 to 0.67
South East Asian	389	3547534	10.97	0.63	0.55 to 0.71	0.51	0.44 to 0.59
Indian	146	982581	14.86	0.85	0.71 to 1.02	0.74	0.60 to 0.91
*Serious injuries*							
Western European	3621	3325410	10.86	1		1	
Eastern European	77	1026072	7.50	0.69	0.54 to 0.88	0.64	0.50 to 0.84
South East Asian	272	3547534	7.67	0.71	0.60 to 0.83	0.56	0.46 to 0.67
Indian	115	982581	11.70	1.08	0.87 to 1.33	0.90	0.70 to 1.16
*Back injuries*							
Western European	82	3325410	2.47	1		1	
Eastern European	8	1026072	0.78	0.32	0.15 to 0.65	0.32	0.16 to 0.67
South East Asian	19	3547534	0.54	0.22	0.13 to 0.36	0.16	0.09 to 0.29
Indian	10	982581	1.02	0.41	0.21 to 0.80	0.50	0.25 to 1.01
*Eye injuries*							
Western European	43	3325410	1.29	1		1	
Eastern European	6	1026072	0.58	0.45	0.19 to 1.06	0.42	0.16 to 1.06
South East Asian	66	3547534	1.86	1.44	0.98 to 2.11	1.25	0.77 to 2.02
Indian	9	982581	0.92	0.71	0.35 to 1.45	0.62	0.26 to 1.46

^a^Adjusted for ship size (> = 3000 vs. <3000 gross tonnage), position (officer vs. non-officer) and age of employee.

The incidence rates for serious injuries showed a similar nationality pattern as that observed for all cases, although the differences are smaller (Figure 1B). Indian seafarers were as frequently seriously injured as Western Europeans, while South East Asian and Eastern European employees have lower rates of serious injuries. A decreasing trend could be noted among Eastern European seafarers and there was also a drop from 2010 to 2011 among Western Europeans. The South East Asian and Indian figures varied over time without any specific trend. The rates of serious occupational injuries for South East Asian and Eastern European seafarers were significantly lower than those of Western European seamen both in crude and adjusted analyses when using combined data (Table 1). The difference between Western European and Indian employees disappeared, even after adjustment for confounding factors, and this finding stood for each year examined, too.

**Figure 2:**
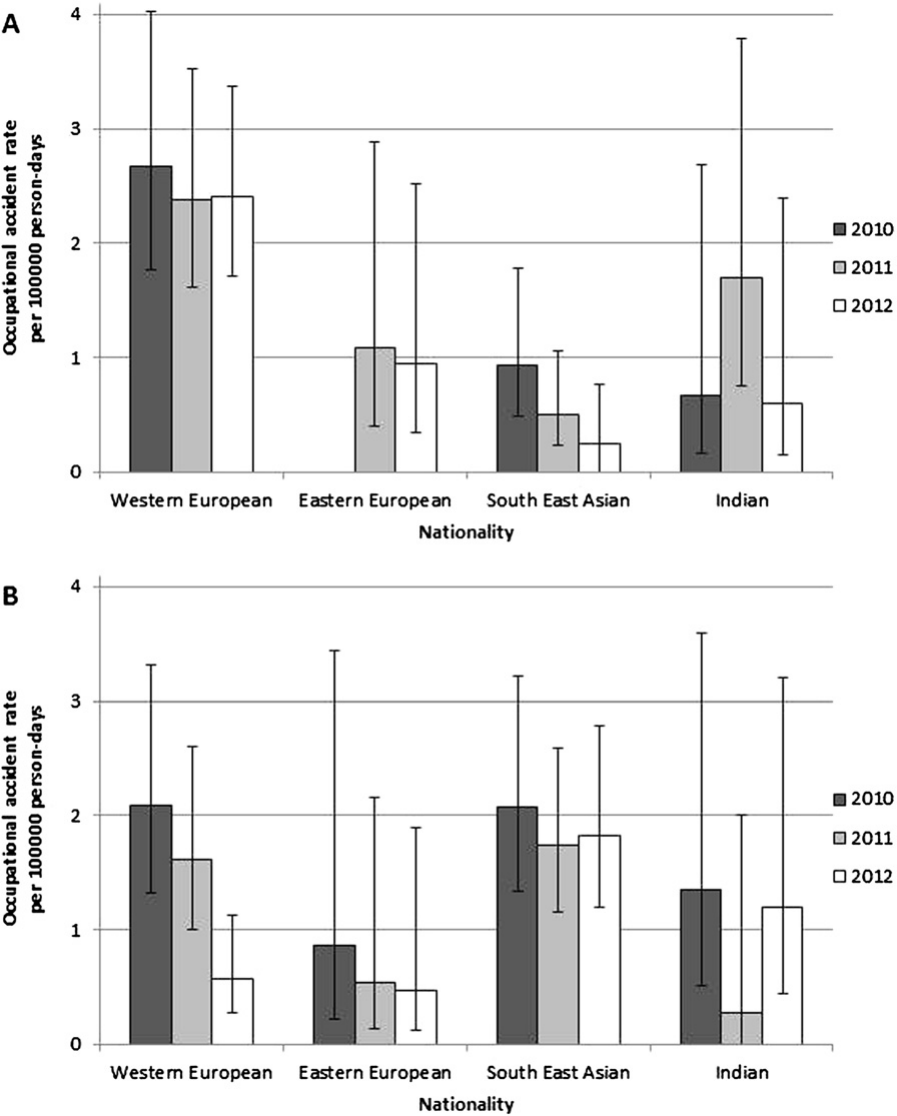
**Accident rate of occupational back (A) and eye (B) injuries reported to the Danish Maritime Authority and to Radio Medical Denmark by nationality of seafarers working on non-passenger merchant ships registered in the Danish International Ship Register.**

An interesting issue was the characterisation of two special types of injuries, to the back and the eyes, respectively. Regarding back injuries, even more pronounced nationality differences were discovered than with all injury types (Figure 2A). With 2.5 injuries per 100000 person-days, Western European seafarers reported back injury by far the most frequently, even after a drop in the rate from 2010 to 2011. The frequency of back injuries for all other nationalities remained much lower, except for Indian employees in 2011. The statistical analysis clearly describes the above findings (Table 1). All nationality groups had significantly lower injury rates than Western European seafarers, except for Indian seamen in the adjusted comparison. The significant differences largely disappeared in the year-by-year analysis, with only South East Asian seafarers reporting significantly lower rate of back injury than Western European workers in each year of the investigated period.

The nationality distribution of occupational eye injury rates shows a different picture than that observed for all injuries, serious injuries, or back injuries (Figure 2B). South East Asian seafarers had the highest rate over the studied time period, while other nationalities had lower values. The injury rate of South East Asian employees was not only the highest among the nationality groups but also stagnated over time, while Western and Eastern European workers experienced a considerable decrease in eye injury rates. South East Asian seafarers had an increased risk of suffering eye damage compared to their Western European colleagues, which was almost statistically significant in the crude comparison (Table 1). When the rates of eye injuries were compared by year, a significantly higher rate among South East Asian seafarers was observed in 2012, which reflects the inverse time trend of the two groups. The injury rates of all other nationality categories were lower but not significantly different form that of Western European workers.

## Discussion

Western European and South East Asian seafarers form the largest groups among seafarers on Danish non-passenger merchant ships and they contribute to the highest number of occupational accidents. The accident rate calculated for the time at risk, that is, the risk of getting injured on board a ship, however, significantly varies among nationality groups. Western European seafarers have a significantly higher incidence rate of reported accidents than colleagues of other nationality backgrounds. This observation highlights that the phenomenon that has been previously reported from Denmark still existed in the 2010-2012 period [7,8], though the accident rate of Western European seafarers shows a characteristic decreasing trend, leading to a reduction in differences over time.

The occupational injury rates reported from different countries vary considerably. This phenomenon is not surprising considering the wide differences in workplace conditions, safety measures, competencies and legislative requirements [9]. Nevertheless, substantial differences relating to employees’ nationality can also be observed within the same country’s workforce where similar standards apply, therefore the divergence cannot be explained by largely differing regulatory backgrounds and workplace conditions. Although seafarers of different nationalities hold different positions and jobs on board, human factors are also likely to play a major role. Previous reports identified typically higher occupational accident rates among foreign workers and some minority groups, although these findings are not consistent [10-14]. Contrary results were reported by a US survey that observed significantly lower risk of occupational injuries among Afro-American and Hispanic than among non-Hispanic white nurses [15]. Zhang et al. also found significantly lower rate of reported work-related injuries among foreign-born workers versus US-born workers [16].

Only few studies exist that investigated the accident rate of seafarers of different nationalities. However, they tend to report consistent findings that seem to contradict some observations reported from other specific workplace settings. Hansen and his colleagues observed that foreign seafarers working aboard merchant ships registered in the Danish International Ship Register had only half the risk of being injured than had a Danish seafarer [7]. A later study by the same author reported an accident rate of 106 per 1000 years at sea among Western European seafarers in 2003, corresponding to 29 injuries per 100000 person-days [8]. Although our investigation was limited to a three-year period, which restricts firm conclusions about trend, the accident rate of 17.5 injuries per 100000 person-days observed for the same nationality group indicates a decreasing trend over the past decade, one that seems to continue in this study period. The study also confirmed the nationality differences, identifying high rates of reported accidents among Western European compared with Eastern European and South East Asian employees [8]. The differences were smaller when only serious injuries were considered, but remained significant. This finding may suggest a certain level of underreporting, but it cannot be the sole explanation. Similar differences between Danish, EU and non-EU nationalities in their reported accident rates were identified recently, based on preliminary analysis of cumulative incidence rates of accidents notified to the Danish Maritime Authority in 2010-2012 [17]. A significantly lower risk of serious occupational injuries among Filipino workers compared to non-Filipinos was also identified in the international crew of a cruise ship registered in the Bahamas [18].

The observed nationality differences in the rate of reported occupational accidents may indicate a difference in the level of risk, or may be an artificial finding due to divergence in reporting. Foreign, especially Asian, seafarers may be reluctant to report injuries, and this could be the reason for their lower rate of notified accidents. A secondary analysis of injury data reported to 16 maritime administrations highlighted the phenomenon of systematic differences between national groups in their propensity to self-report injuries and demonstrated that this can at least partly explain the variations in their injury rates [19]. The typical reasons for under-reporting, as observed in the construction industry, are the perception of the injury as part of the job and the fear of negative consequences of reporting [20]. Workers are more likely to report serious injuries, since these cases frequently require medical treatment and therefore leave less room to conceal the event. The differences found in the rate of serious injuries between nationality groups were found smaller in this study than the differences in overall injury rates. Still, the rate of serious injuries of South East Asian and Eastern European seafarers remained significantly lower than that of Western European seamen. Nevertheless, the significant difference disappeared for Indian employees. The reduced discrepancy indicates the influence of reporting on accident rates but the remaining significance suggests that it is not the only contributing factor.

The study considered an injury serious if medical treatment was sought or recommended for a case or if it was reported as a lost time accident. Although widely used, some ambiguity of categorising an accident as LTA in the special working environment of ships must be noted. The common understanding of being away from work is challenged when the place and activities on duty and off duty easily overlap with each other as frequently experienced in the confined community of a ship. The current manning of merchant ships is limited and everybody is needed for the required tasks and shifts on board, so it is very rare for somebody to stay away from work entirely even if injured. This phenomenon could be the explanation for the large proportion of Radio Medical contact that was not notified to the DMA.

The risk of occupational accident is determined by the specific work environment and the work performed. Therefore the type of the ship as well as the position of the seafarer essentially influences the chance of getting injured [7]. Accidents are typically more frequent among manual than among white-collar workers, that is, in the maritime context, among non-officers compared to officers [5,19]. On Danish ships, the majority of officers are Danish and other Western European seafarers, while Eastern European, and especially Asian, workers typically take lower ranking positions. The potential confounding effect of the uneven distribution of these characteristics among the nationality groups was adjusted for in the regression analysis.

The study defined Indian seafarers as a separate nationality group. Previous studies typically categorised Indian employees as Asians together with Filipinos, or placed them in the “other” nationality group. Therefore until now there has been a lack of data on their maritime safety status. Our findings show remarkable differences in the occupational accident rates of Indian and South East Asian (Filipino) seafarers, with Indians being injured more frequently. Their rate of sustaining a serious injury even proved to be on the level of Western European employees. It indicates that after mitigating the effect of differences in reporting, the risk level of Indian workers does not seem to be different from that of Western European seamen.

Especially large differences were identified among nationality groups in terms of back injuries. For this injury type the rate of Western European seafarers proved to be far higher than that of all other nationalities. Lower rates of acute low back pain were similarly observed among Asians when compared to other ethnic groups in the American population [21,22]. The phenomenon is difficult to explain. Apart from differences in fitness to work, awareness of the seriousness of the problem and the consequent willingness to report it, differences in habits of positioning the trunk and techniques of lifting loads can be hypothesised. The cultural background may determine the individual perception of low back pain, too [23].

Surprisingly the nationality pattern of eye injury rates conflicted with the pattern of other injury types. The rate of eye injuries among South East Asian seafarers proved to be the highest, almost significantly higher than the rate of Western European employees in crude comparison. Further studies are needed to explore the causes behind this observation, which may be due to the special tasks undertaken by Asian crew.

Even though South East Asian seamen proved to have a lower accident rate than Western Europeans, one notable observation is that their rate remained at the same level during the study period, while the Western European seafarers’ rate decreased. This finding indicates an improvement in their safety of Western European seamen, while the safety of South East Asian employees stagnated. So on the one hand Filipino seaman are “better” as regards safety performance while on the other hand their safety is not improving further. Does this mean that they reached their limit of safety? It may be worth exploring this speculative question in further research.

## Conclusions

The study demonstrated substantial differences between nationalities in occupational injury rates reported from merchant ships sailing under the Danish flag. The results confirm previous findings but also highlight decreasing differences over time. The significantly higher rate among Western European seafarers can only partially be related to reporting practice, and the reasons behind it are likely to include differences in safety behaviour and fitness to work. The varying safety culture of nationality groups in the crews of Danish merchant ships has already been suggested and discussed [24]. The effect of national culture on safety behaviour has also been demonstrated among seafarers of different Asian nationalities [25]. Nevertheless, further research is needed to explore the underlying causes of nationality differences in occupational safety culture so as to gain information that can be utilised favourably to eliminate the factors determining potential unsafe behaviour in the hazardous working environments on board merchant ships.
